# Considerations for establishing and maintaining international research collaboration: the example of chemotherapy-induced peripheral neurotoxicity (CIPN)—a white paper

**DOI:** 10.1007/s00520-023-08301-5

**Published:** 2024-02-12

**Authors:** Paola Alberti, Andreas A. Argyriou, Jordi Bruna, M. Imad Damaj, Sara Faithfull, Alice Harding, Ahmet Hoke, Robert Knoerl, Noah Kolb, Tiffany Li, Susanna B. Park, Nathan P. Staff, Stefano Tamburin, Simone Thomas, Ellen Lavoie Smith

**Affiliations:** 1 University of Milano-Bicocca, School of Medicine and Surgery, Monza, Italy; 2 Fondazione IRCCS San Gerardo dei Tintori, Monza, Italy; 3 Neurology Department, Agios Andreas General Hospital, Patras, Greece; 4 Hospital Universitari de Bellvitge, Neuro-Oncology Unit, Institut Catala d’Oncologia (IDIBELL), L’Hospitalet del Llobregat, Barcelona, Spain; 5 Department of Pharmacology and Toxicology and Translational Research Initiative for Pain and Neuropathy, Virginia Commonwealth University, Richmond, VA USA; 6 Trinity College Dublin, School of Medicine, Dublin, Ireland; 7 University of Dublin, Trinity Centre for Health Sciences St. James’s Hospital Campus, Dublin, Ireland; 8 University of Alabama at Birmingham, Office of Sponsored Programs, Birmingham, AL USA; 9 Department of Neurology, Johns Hopkins School of Medicine, Baltimore, MD USA; 10 Department of Health Behavior and Biological Sciences, University of Michigan School of Nursing, Ann Arbor, MI USA; 11 Department of Neurological Sciences, University of Vermont Robert Larner College of Medicine, Burlington, VT USA; 12 Faculty of Medicine and Health, University of Sydney, Brain and Mind Centre and School of Medical Sciences, Sydney, Australia; 13 Department of Neurology, Mayo Clinic, Rochester, MN USA; 14 Department of Neurosciences, Biomedicine and Movement Sciences, University of Verona, Verona, Italy; 15 Department of Neurology, Johns Hopkins School of Medicine, Baltimore, MD USA; 16 Department of Acute, Chronic & Continuing Care, University of Alabama at Birmingham School of Nursing, Birmingham, AL USA

**Keywords:** International collaboration, Chemotherapy-induced peripheral neurotoxicity, Research

## Abstract

**Purpose:**

This white paper provides guidance regarding the process for establishing and maintaining international collaborations to conduct oncology/neurology-focused chemotherapy-induced peripheral neurotoxicity (CIPN) research.

**Methods:**

An international multidisciplinary group of CIPN scientists, clinicians, research administrators, and legal experts have pooled their collective knowledge regarding recommendations for establishing and maintaining international collaboration to foster advancement of CIPN science.

**Results:**

Experts provide recommendations in 10 categories: (1) preclinical and (2) clinical research collaboration; (3) collaborators and consortiums; (4) communication; (5) funding; (6) international regulatory standards; (7) staff training; (8) data management, quality control, and data sharing; (9) dissemination across disciplines and countries; and (10) additional recommendations about feasibility, policy, and mentorship.

**Conclusion:**

Recommendations to establish and maintain international CIPN research collaboration will promote the inclusion of more diverse research participants, increasing consideration of cultural and genetic factors that are essential to inform innovative precision medicine interventions and propel scientific discovery to benefit cancer survivors worldwide.

**Relevance to inform research policy:**

Our suggested guidelines for establishing and maintaining international collaborations to conduct oncology/neurology-focused chemotherapy-induced peripheral neurotoxicity (CIPN) research set forth a challenge to multinational science, clinical, and policy leaders to (1) develop simple, streamlined research designs; (2) address logistical barriers; (3) simplify and standardize regulatory requirements across countries; (4) increase funding to support international collaboration; and (5) foster faculty mentorship.

## Introduction

While cancer survivors have benefited from steadily improving overall survival rates and quality of life, most of the estimated 18 million cancer survivors in the United States (US) alone experience acute and/or long-term toxicities following cancer treatment [1]. Chemotherapy-induced peripheral neurotoxicity (CIPN) is a common consequence of neurotoxic cancer treatment. Despite decades of research, discovery of effective interventions that prevent/mitigate neurotoxicity has been limited. It is becoming increasingly evident that collaborations both nationally and internationally are necessary [2, 3], yet we found no published recommendations in this regard. Documented barriers to effective international collaboration in research, particularly for therapeutic trials, include lack of funding [2–4], lack of time for research [2–4], discordant regulatory requirements among participating countries [2–4], insurance requirements, contractual issues, translation of patient-facing materials into the native language, drug supply, biospecimen collection and shipment, data sharing [4], and the fact that the majority of trials are led by high-income countries [5]. What follows is an in-depth discussion of these barriers and recommendations for addressing them.

## International collaboration in preclinical CIPN research

In the absence of an efficacious CIPN treatment [6], preclinical studies are warranted to provide a strong biological rationale for clinical trials. Numerous CIPN models, both in vitro and in vivo, have been described in the literature [7], but these models may not reproduce the full spectrum of CIPN. International research teams should concur about preclinical experimental research methods that will optimize clinical translation and generalizability to diverse populations. As an example, when conducting in vivo research, scientists must carefully select from several in vivo animal models (mostly in rodents) that can be used to study CIPN [8]. Further, short-term and low-dosage treatment schedules should be avoided if the final aim is to reproduce the full CIPN spectrum [9]. Moreover, to verify that CIPN has ensued, multimodal assessments should quantify behavior, neurophysiology, and histopathology related to both unmyelinated and large myelinated fibers [8–10].

Animal studies that explore CIPN mechanisms should be conducted in accordance with national and international regulatory guidelines that are consistent with 3R principles (replacement, reduction, and refinement) [11]. Replacement refers to using methods that avoid using animals when possible, such as using secondary data from previously conducted studies, human tissue, or established cell lines. When animal studies are necessary, attention to reduction methods will ensure that scientists use the smallest number of animals that are necessary to adequately address the research question without compromising scientific rigor. Sample size calculations should ensure that an appropriate but parsimonious number of animals are used. Lastly, refinement refers to the importance of using methods to minimize pain and distress and ensure animal welfare. For example, if seeking preliminary data regarding the efficacy of an oral agent to prevent CIPN, when possible, the agent should be administered to the animals via their usual food, instead of via oral gavage methods that cause increased distress. Further, to optimize the translational application of findings from animal studies to humans, the age, sex, and genetic homogeneity/heterogeneity of animal models should match, as closely as possible, the target human population. Aligning animal model predictor variables to those seen in humans is important because CIPN onset, severity, and chronicity outcomes can be confounded by these variables. For example, CIPN severity is positively correlated with older age, hormonal variability based on sex can modify underlying pain processing mechanisms, and unique genetic polymorphisms can predispose an animal or human to more or less severe CIPN. Notably, scientists should also perform non-interference studies to assess CIPN intervention interference with chemotherapy drug efficacy, relying on tumor-bearing animal models [12].

To overcome barriers to international collaboration in preclinical CIPN research, scientists must identify funding that can foster such collaborations (governments, intergovernmental groups such as the United Nations, scientific organizations, philanthropic groups). Further, scientific organizations should promote international collaborations.

## International collaboration in clinical CIPN research

The standard challenges of a multidisciplinary and multisite collaborative clinical protocol are amplified by additional CIPN-specific challenges, such as differences in languages, culture, genetics, and country-specific regulations. A critical requirement is to reach agreement on the research protocol: background and rationale, objectives, study design, inclusion/exclusion criteria, assessment of efficacy and safety, and statistical considerations [13]. Designing interventional studies is even more complex, because any drug administered to manage neurotoxicity, either preventive or curative, must not compromise the chemotherapy drug’s anti-cancer efficacy. For this reason, the US Food and Drug Administration (FDA) mandates sound biological evidence that concurrently administered pharmaceutical interventions to manage negative treatment effects can be given safely alongside the chemotherapy drug(s) [14].

The choice of neurotoxicity assessment tools (e.g., patient-reported outcome [PRO] measures, biomarkers, neurological exams, neurophysiological tests, functional assessments) is a crucial and likely the most contentious point to consider when collaborating to design international neurotoxicity studies. Importantly, if CIPN is the primary endpoint of the study, no consensus exists on which measures should be used as the primary outcome assessment [15]. Neurotoxicity assessments may include PRO measures, clinician-rated scales (Cli-RS), and functional or biological biomarkers, all of which will inform variable sample size calculations. Suboptimal correlation between PROs and Cli-RS [16] and clinicians’ variable interpretations of patients’ symptom severity and burden can make it difficult to compare results across studies [17]. Further, not all widely used measurement tools have been translated and validated in all majority languages, such as Spanish or Chinese [18]. Further, cross-cultural differences in symptom interpretation pose challenges, as does the need for feasible (e.g., remote) scientist and staff training to ensure strong inter-rater reliability of clinician-based assessments.

While PROs may be the primary outcome, additional objective measures (e.g., neurophysiological tests) are desirable as secondary endpoints, and may offer hints on the underlying pathophysiology of toxicities [19]. From this perspective, biomarkers (e.g., neurofilament light chain [NfL]) represent a promising higher-sensitivity proxy of early damage [20], even before neurotoxic symptoms arise. Further, in selected settings, innovative tools, such as wearables, sensors, and other telemedicine devices, might be explored as secondary outcome measures to record surrogate measures of toxicity at home, avoiding frequent outpatient visits [21]. Lastly, logistical considerations are required; neurotoxicity assessments may be difficult to access in resource-limited settings and should be planned to minimize participant burden.

## Identifying collaborators and building consortiums

Strong international collaborations can maximize scientific impact by exploiting large consortiums whose members represent diverse expertise and perspectives, including patient advocate representation. In the CIPN field, the Multinational Association of Supportive Care in Cancer (MASCC), Peripheral Nerve Society (PNS), American Society of Clinical Oncology (ASCO), American Society of Preventive Oncology (ASPO), European Society for Medical Oncology (ESMO), and Oncology Nursing Society (ONS), among others, are consortiums that can facilitate clinical research collaboration. Since numerous research techniques and approaches are strongly suggested in preclinical research, another strategy is to build a CIPN-focused international research consortium that fosters scientific rigor and facilitates collaboration and consensus among preclinical and clinical CIPN scientists. All members would agree on the scientific focus and approach via a mutually agreed-upon protocol.

A consortium could also foster rigorous and impactful clinical research studies that employ feasible outcome measures with strong clinimetric properties (e.g., reliability, validity, sensibility, specificity, responsiveness). One strategy is to design a consortium-based *core study* that can be carried out worldwide, using low-cost and easy-to-administer outcome measures. An *extended study* could be performed at centers able to collect more data (e.g., neurophysiological tests, biological specimen collection). In the past, this strategy was successfully applied to the CI-PeriNomS study [22] and is currently ongoing for the International CIPN Assessment and Validation Study (ICAVS, Study Record | ClinicalTrials.gov) that is focused on CIPN assessment.

## Communication

Efficient communication and dissemination of research findings is of utmost importance and a hallmark of successful multinational scientific collaboration and cohesion [23]. Attending conferences and participation in virtual meetings arranged by special interest groups to discuss new or ongoing research projects can further cement international collaboration. However, travel restrictions, language barriers, and health or funding restrictions may significantly restrict in-person participation in international conferences. Indeed, virtual attendance at international conferences is now quite feasible due to the use of remote teleconference technology, shared drives, and open-access websites [24]. However, despite the recent increase in virtual meeting attendance opportunities, numerous barriers to virtual communication approaches, such as discordant time zones, still exist.

Research teams should employ creative strategies for overcoming all of the aforementioned barriers. Such strategies include (1) sharing meeting agendas and other written documents in advance to provide sufficient time for reading and comprehension, and the opportunity for comment; (2) recording meeting minutes; (3) sharing meeting recordings; (4) if feasible, organizing meeting times to enhance participation across time zones; and (4) disseminating information via newsletters and/or social media. Another aspect to be considered is communication to diverse constituents such as patient advocacy groups, government organizations in charge of health policies, and industry partners (e.g., pharmaceutical companies).

## Funding for international research collaboration

To conduct cutting edge research in the CIPN field, research funding is required to support multicenter preclinical and clinical research collaboration. To strengthen the competitiveness of CIPN grant applications for broad oncology or neuroscience funding programs, CIPN researchers must not only disseminate their findings within the scientific community, but also to patients and other constituents to increase awareness of CIPN’s link to clinically important adverse outcomes. Moreover, building consortiums that are inclusive of diverse thinkers and disciplinary experts will foster innovation that could be pivotal in securing funds. The Toxic Neuropathy Consortium of the PNS [25] and the Neurological Complications Study Group of MASCC might provide fundamental infrastructure in this regard.

While funding from large national funding bodies (e.g., National Institutes of Health [NIH], the US Department of Defense, or Cancer Research UK) is desirable because these organizations can support larger and more impactful research studies, grant funding from these organizations is difficult to obtain, and some do not allow fund allocation to international entities. As an alternative approach, grant submissions to neuropathy-focused foundations or multinational scientific organizations could increase the likelihood of obtaining funding for international CIPN research. These foundations include the Foundation for Peripheral Neuropathy, the American Cancer Society, MASCC, and the European Cancer Organisation. Furthermore, yearly calls opened by the European Research Council might be appropriate since they allow submissions regarding any topic and often allow collaborations with US research institutions.

After identifying a viable funding mechanism, unique nuances in how a grant is crafted may pose barriers to writing a highly competitive application. When working collaboratively to develop a research budget, international collaborators should consider variations in how research activities are funded in countries with a national health care system versus an insurance-based system (e.g., Italy versus the US). In some countries, research-based assessments and researchers’ time are paid for by the hospital, and thus ancillary funding for these activities is not required. However, the costs associated with research studies that are conducted in the US cannot be billed to insurance companies, necessitating additional funding to pay for researcher and staff salaries and all procedures performed during the study that are not standard of care*.* Thus, the cost of doing research in one country versus another will influence the scope of the proposed international research that will be feasible for all participating centers.

## Human subject protections, international variation, and contracts

Each institution handles contracting differently, making it difficult for international partners to co-create universally applicable guidance. Understanding the differences in human subjects’ protection regulations from country to country is a first step.

For example, the toughest privacy and security law in the world is the European Union’s General Data Protection Regulation (2106), or GDPR. Although it was drafted and passed by the European Union (EU), it imposes obligations that *all* participating countries must adhere to if the research will be conducted with people in the EU [26]. The difference between the GDPR’s stricter regulations and the guidelines imposed by the US’s Health Insurance Portability and Accountability Act (HIPAA) (1996) is a major barrier to international research collaboration. Modification of US requirements to align with those of the GDPR cannot be easily accomplished because US research enterprises have spent two and a half decades ensuring that their systems are complaint with HIPAA and newer data security laws, such as the Children’s Online Privacy Protection Act (1998) and the Gramm-Leach-Bliley Act (1999). Further, individual states have unique laws like the California Consumer Privacy Act (2019), California’s Consumer Privacy Act (2023), and the Virginia Consumer Privacy Act (2023) [27]. Receipt of funding for international research collaboration requires updated US data security systems that can be compliant with the many different regulatory requirements. Without the appropriate technology infrastructure, US data recipients are unable to ensure compliance with foreign data privacy regulations and, increasingly, domestic protections. Because regulations will vary by country, it is important to review the laws for the country in which the international partner is situated.

Flexibility and a willingness to negotiate terms outside of regular data protection regulations may be key to successful collaborative agreements. A new trend is to require research partners to click a catch-all tab on a website that obligates the partner to comply with all current and future regulations. The full volume of laws, regulations, and rules may be difficult to assess, as information can be in seemingly endless dropdown menus that link to page after page of information, sometimes embedded with even more links. For US institutions that are accustomed to reviewing a static contract, this can become burdensome, or even impossible to send through the normal review process. Not all research partners will be able to agree to all foreign legislation. For example, an institution that is unable to comply with the Declaration of Helsinki (1964) may suggest the alternate Belmont Report (1978); however, unwillingness to allow these sorts of changes can result in a complete breakdown in international negotiations.

Corporate culture also varies and may inhibit negotiations. US institutions may find that European contracts contain language mandating investigator development, continuing education, and even career ladders that do not exist at the US institutions, underscoring differences in career development. In Europe, the institution has a hand in the process; in the US, researchers drive their own career development and may change employers to seek higher-level opportunity. If a US institution is unable to modify the language, it is likely that negotiations will fail. Lastly, a contract with an international partner may not be long enough or lucrative enough to motivate a US institution to make sweeping changes that are outside the norm in the States but would be in line with the European standard.

Updates in systems and data security should be done with consideration for compliance with GDPR and the knowledge that international collaboration will require this. It is critical to these efforts that institutions are mindful of the need to be open to contract negotiation and willing to appreciate the differences in how we all do business and the variations in mandated regulations.

## Staff training, manuals, and standard operating procedures

One area of great concern in international multisite studies is the training and qualification of staff members. It is important to identify the training requirements for each position, provide access to the required training, and verify that each staff member meets the qualification criteria after training is complete [28]. In-person, hands-on instruction is usually more efficient and effective than online training modules, as it allows more flexibility to adjust the training to the level of the trainees’ knowledge and permits confirmation by the instructor that the trainees achieved proficiency. It also provides opportunities for the trainees, particularly novice non-medical personnel, to ask questions to increase conceptual understanding [29]. Most senior personnel are proficient in English, as this is the common language in research; however, proficiency in English cannot necessarily be expected in staff members, and training might be most beneficial if provided in the native language. If in-person training is not feasible, online training modules and videos are acceptable alternatives, as long as knowledge and skill proficiency is confirmed by the site’s principal investigator (PI) or other senior personnel. Verification that staff members are qualified to administer an assessment is critical [30], as data is only reliable when testing is conducted in a standardized manner. Besides training, another efficient and effective way to ensure uniformity among study sites is through detailed written instructions.

Another critical step to ensure uniformity in data collection among sites is the preparation of Case Report Forms (CRFs) together with checklists, as this is the most efficient way to ensure that all required data are collected during research visits. Providing Standard Operating Procedures (SOP) as reference material to study staff further helps to communicate desired knowledge [31]. While manuals detail the testing procedures, SOPs provide instructions on how the test results should be converted into data, which reduces data errors due to lack of medical knowledge.

Once research is underway, it is important to provide a forum for scientists and staff members to reach out for advice, clarify procedures, and raise problems for group brainstorming. Regular research team meetings at each participating site, as well as multinational webinars that connect all participating sites together and are led by the project PI(s), provide forums for information sharing while the project is ongoing. Further, a designated point of contact will allow site personnel at any site to obtain answers to emerging questions.

## Data management, quality control, and data sharing

Data management and quality control strategies ensure high-quality data by keeping errors, missing data, and arbitrary data variation among raters and sites as low as possible [32]. The best practice is to prepare in advance a data management plan that details the database design, data entry and tracking guidelines, quality control measures, discrepancy management, data validation, and database locking guidelines. The Society for Clinical Data Management (SCDM) provides information for both minimum standards and good clinical practices regarding data management [33].

The most important tasks in the clinical data management process are designing and annotating CRFs, creating the database, entering and validating the data, managing inconsistencies and resolving data disputes, medical coding, data extraction, database locking, documenting data management processes, and providing data security [34]. The most important step to ensure reliable and high-quality data is validation of the data immediately after it is collected by means of database queries and record screening by an expert reviewer to detect missing data points, data entry errors, and data inconsistencies. However, these steps are often neglected in research that does not receive commercial funding [35].

In recent years, it has become common practice to share data from clinical trials with third parties for additional research applications. When doing this, the data set should be modified to meet the standards of a de-identified data set for this purpose; data requests are usually processed following peer review [36]. Written Data User Agreements (DUAs) should be prepared to detail citation and authorship expectations, publication rights, guidelines for storage of the data, and the process for additional analysis projects.

## Dissemination strategies across disciplines and countries

Diffusion theory, a model used to understand how medical knowledge is disseminated and adopted [37, 38], posits that learners (e.g., researchers, physicians, advanced practice providers) typically learn about innovations and new data through mediated content (e.g., posters and presentations at conferences), then utilize sources they trust to validate the information and make decisions about implementation. This concept is recognized by the NCI as an important implementation strategy to spread new knowledge [38].

To enhance dissemination of new findings, major national and international organizations should partner with research teams to distribute knowledge broadly utilizing diffusion theory. To ensure equitable opportunity for meeting attendance, it is important for planning committees to take steps to ensure that meeting locations are equitable and not concentrated solely in the same global regions annually. One unexpected yet positive outcome of the COVID-19 global pandemic is increased utilization of virtual platforms for international meetings. In resource-poor areas and for practitioners whose primary responsibility is patient care, online access to novel research data is a large step towards leveling the playing field and providing equitable access. Consequently, it is important that, within the major international organizations, access to online content is considered, maintained, and prioritized in advance of annual meetings.

Historically, research participation and dissemination of the findings of collaborative projects are skewed towards research-rich countries. The rapid spread of social media is changing the paradigm for the dissemination of knowledge and is free of charge. Social media can serve as a first point of contact to spread awareness, then learners can further pursue knowledge by reading related publications. It is important to point out that, to maintain scientific integrity, the peer review process is still mandatory. However, a clear benefit of social media is that, in addition to academics and clinicians, it engages a broader audience (including patients) in an informal way [39]. Organizations involved in CIPN research can utilize these dissemination strategies. A current example of this is the work of the PNS’s primarily CIPN-focused Toxic Neuropathy Consortium, which has a subcommittee dedicated to establishing a social media presence and disseminating interesting, important, and emerging research. Because the role of social media is increasing and changing over time, further work is needed to understand how best to use these platforms to ensure broad and equitable global dissemination of study findings.

It is important to consider additional alternative methods of disseminating research when available. Podcasts, webinars, and town halls are increasing in frequency and utilization. Specifically, scientific podcasts steadily and consistently increased in the early 2000s with an exponential growth from 2010 to 2018 [40]. Continued growth has expanded scientific audiences, bringing in a diversity of languages and geographic regions [41]. Clearly, pitfalls exist in this new landscape as the peer review process may be limited and listenership can be increased through click bait; however, use of these strategies can expand distribution of research findings worldwide.

Despite the increasing use of new media to disseminate knowledge, the reality is that results are most often published in a single journal or presented at a single meeting with a limited audience. Consequently, research results can be effectively siloed into a single specialty or subspecialty despite having broad applicability and interest. Dissemination across specialties is important as cross-pollination facilitates collaboration among experts with differing skill sets working on similar research questions. In international multi-specialty research groups, investigators may consider presenting abstracts and publishing results in venues that appeal to multiple specialties or spreading the multiple publications from a single group to journals with differing target audiences.

Lastly, as international teams increasingly tackle complex problems globally, data sharing is critical to ensure transparency and reproducibility. A cross-sectional study of cancer-related publications in 2019 found that only 16% of studies made data available to the public, and less than 1% were in compliance with the Findability, Accessibility, Interoperability, and Reusability (FAIR) principles for scientific data management and stewardship, which prioritizes making data findable, accessible, interoperable, and reusable [42]. Interestingly, policies from publishers to encourage data sharing did not appear to affect whether data was actually available [43]. Moving forward, it is important for journals and their editors to be more deliberate about enforcing data-sharing policies and adhering to guidelines that would improve usability [43].

## Additional recommendations: feasibility, policy, and mentorship

Table 1 outlines the authors’ recommendations for establishing and maintaining international collaboration in CIPN research. These include streamlining preclinical and clinical research study design (e.g., use of single Institutional Review Boards [IRB]), simplifying and standardizing regulatory requirements to decrease burden for investigators and participating institutions across countries, and increasing funding for international research collaboration [4]. Additional literature suggests that connecting investigators with international mentors or research networks may be another strategy to foster international research collaboration in oncology [2] and to improve communication and collaboration among multinational constituents to tackle logistical barriers [44].

**Table 1:** Recommendations for establishing and maintaining research collaboration, methodologic consistency, and international translation of CIPN research findings

Collaborate to Conduct Pre-Clinical Research	
1. Reach consensus among collaborators regarding in vivo models and statistical design.* 2. Identify funding sources that foster international collaboration. 3. Encourage scientific organizations to promote international collaboration.	
Collaborate to Conduct Clinical Research	
1. Reach consensus among collaborators regarding the study design and methods.* 2. Provide evidence that the CIPN intervention will not interfere with chemotherapy efficacy. 3. Select outcome measures that have been validated in diverse languages, align with the study aims, and minimize participant burden.* 4. Consider cultural variations in symptom interpretation.* 5. Provide standardized training in outcome measurement to scientists and staff via remote methods.*	
Identify Collaborators and Build Consortiums	
1. Exploit large consortiums to leverage collaborations. 2. Engage partners with diverse expertise and perspectives, including patient advocates. 3. Create international research consortiums that focus solely on CIPN science. 4. Develop a consortium-based core study that can be conducted worldwide.	
Communication	
1. Attend multinational conferences (in-person or remotely) when possible. 2. Join CIPN-focused special interest groups. 3. To address language barriers, share meeting agendas and materials with collaborators before the meeting date to allow sufficient time for reading and comprehension. 4. Record and share meeting minutes and video recordings. 5. Consider meeting times that enhance participation across time zones. 6. Disseminate information via newsletters and/or social media. 7. Share information with diverse constituents (e.g., patient advocacy groups, government organizations, industry).	
Obtain Funding	
1. Strengthen competitiveness of grant applications by obtaining diverse feedback from innovative thinkers. 2. Consider grant submissions to large national funding bodies, CIPN-focused foundations, and multinational scientific organizations. 3. Consider variations in policies mandating how research can be funded in different countries (e.g., grants, national health care systems).	
Human Subject Protections, International Variations, and Contracts	
1. Understand the differences in human subjects protection regulations across countries, with European standards generally being more stringent than those in the US. 2. Consider using a single Institutional Review Board. 3. Review the laws for the involved country, since regulations vary internationally. 4. Try to flex and negotiate the data protection terms. 5. Plan on a US institutional legal review timeline for multinational research contracts. 6. Recognize that a US institution may be unwilling to change policies to align with the European standard. 7. Recognize that updated US data security systems may be needed to comply with European standards.	
Staff Training, Manuals, and Standard Operating Procedures	
1. Identify training requirements for all involved scientists and staff.* 2. Provide standardized hands-on and/or online training and written instructions. In-person training is preferred.* 3. Verify that training has been completed.* 4. Provide training in the trainee’s native language; English proficiency cannot be assumed.* 5. Create Case Report Forms, written Standard Operating Procedures, and process checklists.* 6. Provide a regular forum for discussion and information sharing within and among all participating sites.* 7. Designate a contact person who can address emerging questions from all sites.*	
Data Management, Quality Control, and Data Sharing	
1. Prepare a data management plan that outlines data entry and tracking, quality control, discrepancy management, data validation, and database locking guidelines.* 2. Abide by institutional standards for sharing de-identified data sets and obtain an approved Data User 3. Agreement (DUA) before sharing data.	
Dissemination Strategies Across Disciplines and Countries	
1. Encourage major national and international organizations to partner with research teams to broadly distribute knowledge utilizing diffusion theory. 2. Ensure that conference planning committees set meeting locations that are equitable and not concentrated solely in the same global regions annually. 3. Leverage virtual platforms for international meetings. 4.Use social media platforms to disseminate information and spread awareness, particularly to benefit low-income countries. 5. Consider alternative dissemination methods, such as podcasts, webinars, and town halls. 6. Disseminate findings to multidisciplinary audiences. Share data.	
Feasibility, Policy, and Mentorship	
1. Streamline preclinical and clinical research study designs. 2. Facilitate multinational collaboration to address logistical barriers.* 3. Simplify and standardize regulatory requirements to decrease burden for scientists and research teams. 4. Increase funding for international research collaboration. 5. Connect investigators with international mentors and research networks.	

*Important for maintaining methodologic consistency and international comparability of research findings

Table 1 also highlights important strategies to maintain methodologic consistency and comparability/translation of findings across different countries. These strategies include (1) reaching prospective consensus among collaborators regarding study aims, design and methods; (2) selecting outcome measures that have been validated in multiple languages, (3) consideration of cultural variations in symptom interpretation, (4) standardized training for all research personnel, (5) ongoing cross-country communication regarding study progress and procedures, and (6) use of standardized and articulated operational procedures for maintaining data quality.

Taken together, the aforementioned recommendations for enhancing international research collaboration can lead to improved internal and external validity of CIPN research through prospective consensus regarding rigorous and internationally translatable research design and methods, and inclusion of more diverse research participants. Consideration of diverse cultural, environmental, and biologic factors through international collaboration is essential to inform innovative precision medicine interventions and propel scientific discovery to benefit cancer survivors worldwide.

## Data Availability

Not applicable
