# Correction to: Considerations for establishing and maintaining international research collaboration: the example of chemotherapy‑induced peripheral neurotoxicity (CIPN)—a white paper

**DOI:** 10.1007/s00520-024-08382-w

**Published:** 2024-02-21

**Authors:** Paola Alberti, Andreas A. Argyriou, Jordi Bruna, M. Imad Damaj, Sara Faithfull, Alice Harding, Ahmet Hoke, Robert Knoerl, Noah Kolb, Tiffany Li, Susanna B. Park, Nathan P. Staff, Stefano Tamburin, Simone Thomas, Ellen Lavoie Smith

**Affiliations:** 1 University of Milano-Bicocca, School of Medicine and Surgery, Monza, Italy; 2 Fondazione IRCCS San Gerardo dei Tintori, Monza, Italy; 3 Neurology Department, Agios Andreas General Hospital, Patras, Greece; 4 Hospital Universitari de Bellvitge, Neuro-Oncology Unit, Institut Catala d’Oncologia (IDIBELL), L’Hospitalet del Llobregat, Barcelona, Spain; 5 Department of Pharmacology and Toxicology and Translational Research Initiative for Pain and Neuropathy, Virginia Commonwealth University, Richmond, VA USA; 6 Trinity College Dublin, School of Medicine, Dublin, Ireland; 7 University of Dublin, Trinity Centre for Health Sciences St. James’s Hospital Campus, Dublin, Ireland; 8 University of Alabama at Birmingham, Office of Sponsored Programs, Birmingham, AL USA; 9 Department of Neurology, Johns Hopkins School of Medicine, Baltimore, MD USA; 10 Department of Health Behavior and Biological Sciences, University of Michigan School of Nursing, Ann Arbor, MI USA; 11 Department of Neurological Sciences, University of Vermont Robert Larner College of Medicine, Burlington, VT USA; 12 Faculty of Medicine and Health, University of Sydney, Brain and Mind Centre and School of Medical Sciences, Sydney, Australia; 13 Department of Neurology, Mayo Clinic, Rochester, MN USA; 14 Department of Neurosciences, Biomedicine and Movement Sciences, University of Verona, Verona, Italy; 15 Department of Neurology, Johns Hopkins School of Medicine, Baltimore, MD USA; 16 Department of Acute, Chronic & Continuing Care, University of Alabama at Birmingham School of Nursing, Birmingham, AL USA

**Correction to: Supportive Care in Cancer (2024) 32:117**

10.1007/s00520-023-08301-5

The correct name should be Nathan P. Staff instead of P. Nathan.

The original article is corrected.

